# SATO (IDEA**S** exp**A**nded wi**T**h BCI**O**): Workflow for designers of patient-centered mobile health behaviour change intervention applications

**DOI:** 10.1016/j.jbi.2022.104276

**Published:** 2023-02

**Authors:** Aneta Lisowska, Szymon Wilk, Mor Peleg

**Affiliations:** a Sano Centre for Computational Medicine, Czarnowiejska 36, Cracow, 30-054, Poland; b Institute of Computing Science, Poznan University of Technology, Piotrowo 2, Poznan, 60-965, Poland; c Department of Information Systems, University of Haifa, Haifa, Israel

**Keywords:** Digital behaviour change intervention (DBCI), Mobile health, Personalisation, Application design, Wellbeing, Cancer

## Abstract

Designing effective theory-driven digital behaviour change interventions (DBCI) is a challenging task. To ease the design process, and assist with knowledge sharing and evaluation of the DBCI, we propose the SATO (IDEA**S** exp**A**nded wi**T**h BCI**O**) design workflow based on the IDEAS (Integrate, Design, Assess, and Share) framework and aligned with the Behaviour Change Intervention Ontology (BCIO). BCIO is a structural representation of the knowledge in behaviour change domain supporting evaluation of behaviour change interventions (BCIs) but it is not straightforward to utilise it during DBCI design. IDEAS (Integrate, Design, Assess, and Share) framework guides multi-disciplinary teams through the mobile health (mHealth) application development life-cycle but it is not aligned with BCIO entities. SATO couples BCIO entities with workflow steps and extends IDEAS Integrate stage with consideration of customisation and personalisation. We provide a checklist of the activities that should be performed during intervention planning with concrete examples and a tutorial accompanied with case studies from the Cancer Better Life Experience (CAPABLE) European project. In the process of creating this workflow, we found the necessity to extend the BCIO to support the scenarios of multiple clinical goals in the same application. To ensure the SATO steps are easy to follow for the incomers to the field, we performed a preliminary evaluation of the workflow with two knowledge engineers, working on novel mHealth app design tasks.

## Graphical abstract

**Figure:**
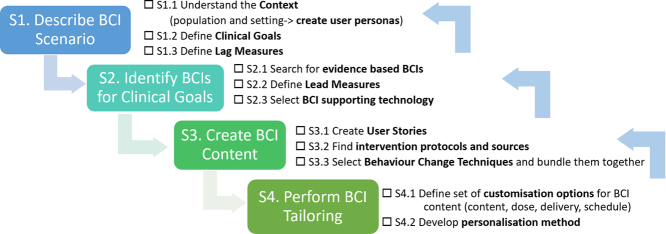

## 1 Introduction

The amount of literature on digital health interventions (DHI) [1] is vast [2], [3], with studies aiming to improve mental [4] and physical health [5]. Interventions cover applications from different stages of the health management cycle and target populations across different ages, social status and cultures. Some DHIs aim to support patients with adherence to pharmacological treatment, dose management and side effects reporting. Other DHIs aid patients with modulation of health risk behaviours such as: inactivity, poor nutrition, or substance abuse. The later are called digital behaviour change interventions (DBCI) and their design, or more specifically design of patient-centered mobile health (mHealth) behaviour change intervention (BCI) applications, is the focus of this paper.

Michie and colleagues have been working for many years on creating a standardisation of the behaviour change domain. The evaluation of BCIs and comparison of theories, studies, and trials that incorporate behaviour change is not feasible without a standard vocabulary and ontology. Such a structure organising the basic techniques that are implemented in BCIs is needed in order to attribute the success of behavioural change to particular behavioural techniques. Michie’s group developed taxonomy of behavioural change techniques (BCT) [6] and the Behaviour Change Intervention Ontology (BCIO) [7]. BCIO is a very useful tool supporting evaluation of interventions’ effectiveness, but it is relatively new and there are only few examples available on how to utilise it in mHealth application development. The previously provided examples do not cover interventions with multiple wellbeing goals and target behaviours. We found that it is not trivial to apply BCIO to that setting. Therefore, in this work we extend BCIO to mHealth applications with multiple wellbeing goals^1^ and provide examples of how to use it during the application development process. Our work aims to facilitate researchers with utilising BCIO for the application design in the future and ultimately ease the interventions’ effectiveness evaluation.

**Fig. 1:**
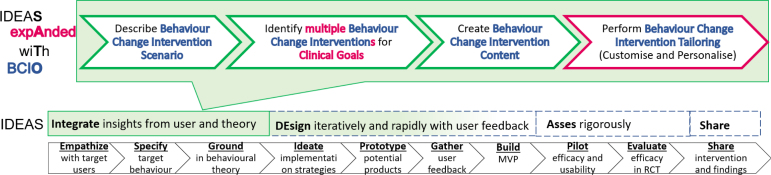
SATO (IDEA**S** exp**A**nded wi**T**h BCI**O**). We expand the Integrate stage of IDEAS and align it with BCIO. Terms from BCIO are marked in blue and our extensions to BCIO and IDEAS are marked in pink.

IDEAS (Integrate, Design, Assess, and Share) [8] is a framework and toolkit of strategies for the development of more effective digital interventions to change health behaviour, integrating methods from behavioural theory, design thinking, and intervention evaluation and dissemination into the full life-cycle of app development. It is popularly used in the development of DBCI and provides a simple and easy to follow checklist of activities that should be performed during DBCI development that might be more accessible to the incomers in the field than the potential more comprehensive design guide provided by Michieet al. [9]. However it was developed in 2016 prior to release of BCIO, therefore, it does not align with it. Rather than discarding the IDEAS framework we show that the Integrate stage could be aligned with BCIO (See Fig. 1). We also extend IDEAS with the tailoring step, which has been previously shown to be a crucial component of effective interventions and mentioned in the Michie et al. [10] recommendations.

This paper intends to provide a detailed actionable systematic workflow for mobile behaviour change application design drawing from IDEAS [8] and in alignment with the BCIO [7] and behaviour change techniques [6]. The SATO (IDEA**S** exp**A**nded wi**T**h BCI**O**) includes a design workflow and checklist (See Fig. 3). We provide examples taken from the Horizon 2020 project Cancer Patient Better Life Experience (CAPABLE, https://capable-project.eu/) [11], where we follow a multi-stakeholder and evidence-based iterative development cycle for a DBCI, in teams of informaticians, clinicians, patients, and engineers, and also utilise feasibility studies on synthetic and public datasets. The main contributions of this research are: (1) application and extension of the BCIO to multi-BCIs mHealth applications that span several clinical goals, (2) DBCI design workflow and checklist validated with examples from the CAPABLE project, (3) reusable design templates bundling together several behaviour change techniques, and (4) tutorial on how to apply SATO to the DBCI application design.

## 2 Related work

We review available DBCI design frameworks and BCIO upon which we base our proposed DBCI development workflow.

### 2.1 DBCI design frameworks

Michie et al. [10] provides recommendations for developing and evaluating DBCIs falling into six general themes: Achieving rapid and efficient development, Understanding and promoting engagement, Advancing models and theories, Evaluating effectiveness, Evaluating cost-effectiveness and Ensuring regulatory, ethical, and information governance. This is a useful starting point for the DBCI developers highlighting all aspects of intervention design, however it lacks demonstration of how the recommendations are applied to a concrete DBCI.

Miller et al. created a framework for Analyzing and Measuring Usage and Engagement Data (AMUsED) in Digital Interventions [12] which focuses on analysis of participant’s effective engagement with the intervention. The accompanying checklist when used during the design stage may help identify which data need to be captured to assess the links between BCTs and behaviour. The authors provide case studies of applying the AMUsED framework to two web-based interventions and suggest that the framework is flexible to fit with interventions delivered with other digital technologies. Although the AMUsED framework is very helpful, especially at the intervention evaluation stages, a broader framework is needed to guide through the full digital behaviour change intervention design process, including content generation and tailoring.

The IDEAS [8] framework guides multi-disciplinary teams through the full mHealth application development life-cycle, and provides a disciplined way to incrementally translate behavioural theories into highly relevant and practical interventions. Its ten steps are organised into a four-phase process: Integrate phase, including (1) empathise with target users, (2) specify target behaviour, (3) ground in behavioural theory; DEsign phase, including (4) ideate implementation strategies, (5) prototype potential products, (6) gather user feedback, (7) build a minimum viable product; Assess phase, including (8) pilot test to assess potential efficacy and usability, and (9) evaluation of the efficacy in an RCT; and Share phase, with (10) share intervention and findings. We previously extended IDEAS with an ontology [13] that structures the target behaviour change intervention as a class derived from the HL7 Fast Healthcare Interoperability Resources (FHIR) standard [14]. We also demonstrated application of the proposed extension to a case study taken from the CAPABLE project, that used Fogg’s Tiny Habits behavioural model [15] to improve the sleep of cancer patients via Tai Chi, delivered via an mHealth app. In another work [16], we extended IDEAS’ Ideate substep of the Design phase, by providing concrete backend architectural components and graphical user-interface designs that implemented behavioural interventions.

### 2.2 BCIO

Ontology is an organisation and representation of the entities in a domain according to their properties and relations to one another [17]. Unlike theories, which provide explanations, onotologies are structural representation of the knowledge [18].

**Fig. 2:**
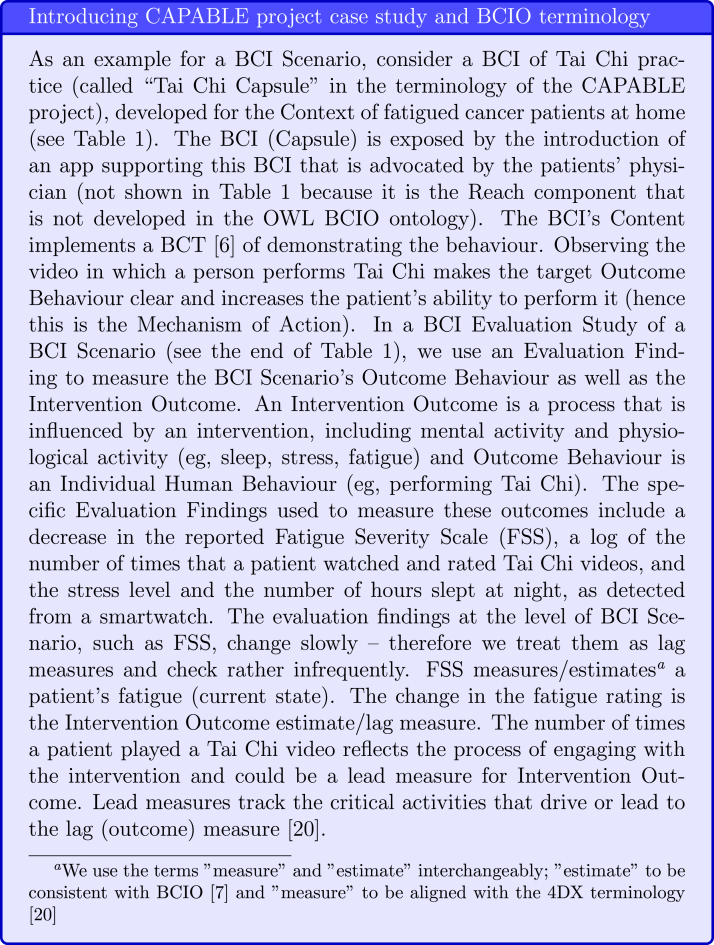
Introducing CAPABLE Project Case Study: “Fatigue reduction scenario” and BCIO terminology.

Michie et al. [7] designed the Behaviour Change Intervention Ontology (BCIO) following the principles of the Open Biological and Biomedical Ontology (OBO) Foundry (https://obofoundry.org) by extending the Basic Formal Ontology [19]. There are six main classes in the BCIO. The (1) BCI Scenario class, in which a (2) BCI, developed for a (3) Context (i.e., target population and setting), is exposed to the population via an (4) Exposure (including the Engagement and Reach activity), and it uses a (5) Mechanism of Action to yield an (6) Outcome Behaviour, which is the intended new behaviour that should form a habit. The Scenario’s Outcome Behaviour can be estimated in a clinical study. The OWL implementation of the BCIO is a work in progress and currently, the Exposure class is not fully developed and only one of its two parts – the BCI Engagement – is defined. Therefore, when using the OWL ontology, we provide an example of Engagement and not of the entire Exposure with its Reach component (see Fig. 2).

**Table 1:**
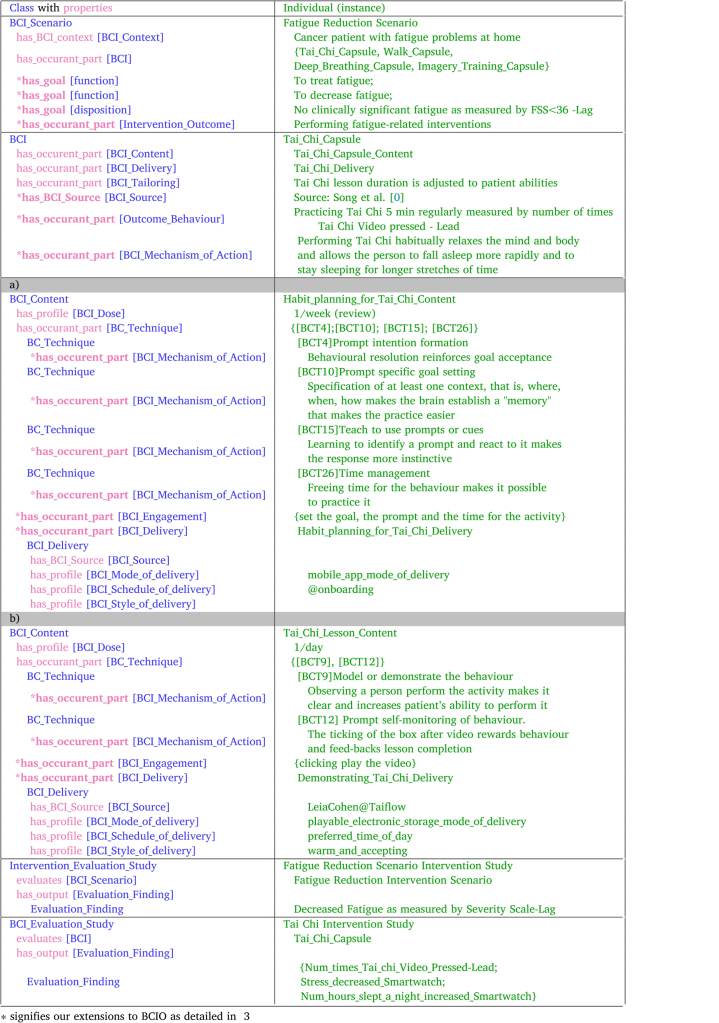
BCIO [7] examples for the Fatigue Reduction Scenario.

**Fig. 3:**
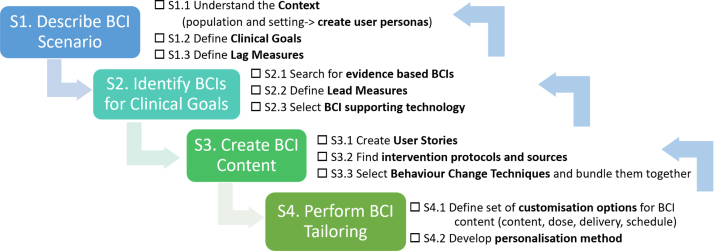
SATO workflow for development of DBCI apps.

## 3 DBCI design steps

An important contribution of this study is the formulation of the SATO (IDEA**S** exp**A**nded wi**T**h BCI**O**) workflow for the development of DBCI apps that create desirable behaviour change. The workflow consist of four main steps (See Fig. 3) that facilitate designers in addressing the following four big questions:

1. What is the clinical problem?
2. What changes in behaviour will support resolution of the problem?
3. How can we facilitate patients with behaviour change?
4. How can we adjust the support to meet the needs of individual patients?

The SATO steps follow BCIO terminology to facilitate utilisation of BCIO in mHealth research and knowledge sharing. SATO is theory-independent, but via BCIO it can support mHealth app design, based on any behavioural theory that the development team selects. To ease understanding of the SATO workflow, we first explain each SATO step and later, in a blue box, provide concrete examples from the CAPABLE project.

**Fig. 4:**
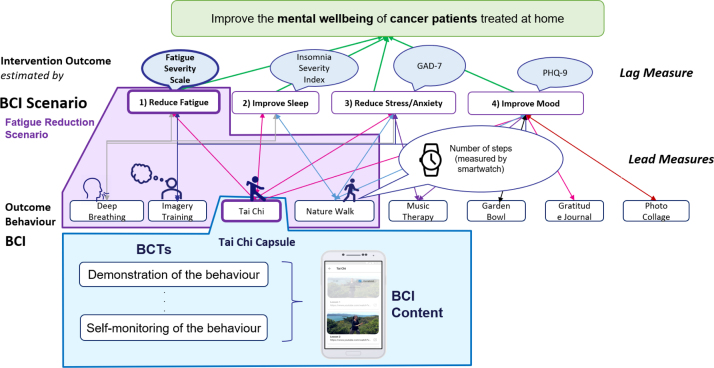
BCI Scenario, BCI, and BCT examples from the CAPABLE project.

We consider the design of a mHealth application that provides multiple clinical goals and for each goal — multiple BCIs. Based on our experience of implementing several clinical goals and BCIs as part of the CAPABLE project, we suggest that each BCI Scenario has a single clinical goal but each app has multiple goals (e.g., to prevent stress, to treat fatigue). Our running example is a BCI Scenario in which the clinical goal “to-treat fatigue”. At the BCI Scenario level, the desired Intervention Outcome is no clinically significant fatigue, as measured by the standard Fatigue Severity Scale [20] and the BCI’s Behaviour Outcome is practice of Tai Chi (Fig. 4).

### 3.1 [S1] Describe BCI Scenario — What is the clinical problem?

Following the IDEAS framework [8], we recommend that the DBCI will be developed by a multi-stakeholder development team consisting of the clinicians whose patients will use the DBCI, the patients themselves, and the technical developers (engineers, informaticians), which would meet regularly during the DBCI development phase. The first step that this team does is defining clinical problem through answering the following questions:

1. Who are the users?
2. What are their goals?
3. How can we measure that they met the goals?

#### 3.1.1 [S1.1] Understand the Context

**Fig. 5:**
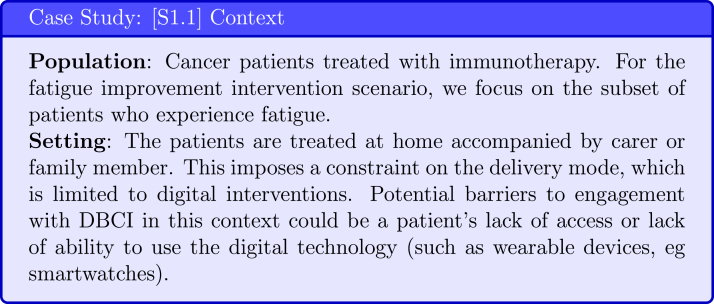
Case Study: BCI Context, (a) BCI population (b) BCI setting including BCI social and BCI physical setting.

Through patient interviews and creation of **user personas** [21] the DBCI design team captures the information about the BCI Scenario’s patient **population** and their **setting** (see Fig. 5). It is important to consider the intervention setting of target users because it determines the possible mode of delivery of the BCI and influences the barriers to engagement. BCIO specifies by both social and physical setting.

**Fig. 6:**
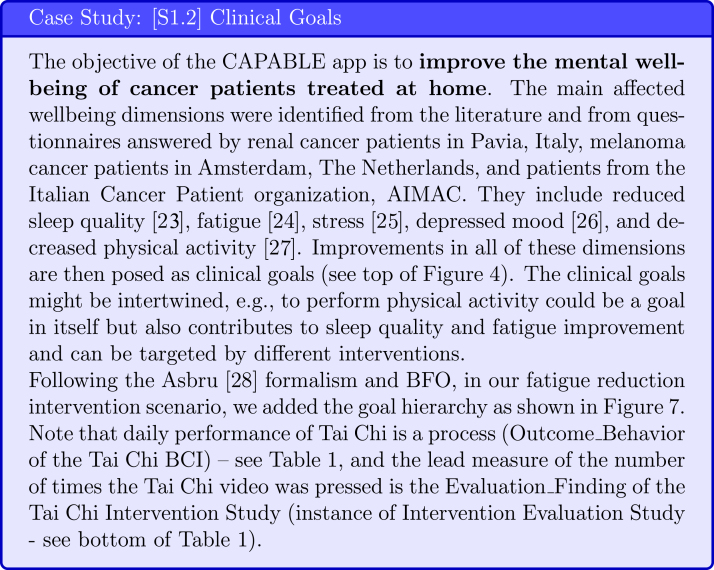
Case Study: Clinical Goals in Capable Project [22], [23], [24], [25], [26], [27].

#### 3.1.2 [S1.2] Identify Clinical Goal (*extension of BCIO)

A crucial step in developing a DBCI is to select the important clinical objective or goal of this DBCI. The clinical goal will impact the choice of intervention and evaluation metrics. To select the clinical goals of the DBCI for the target population, research should be done to establish what wellbeing dimensions [28] are the ones most impacted by the patients’ condition (see Fig. 6), fitting with IDEAS’ Empathise step of the Integrate phase, that integrates insights from users and theory. This step should be led by the clinical researchers and performed via a literature search supplemented by questionnaires applied to the target population.

**Goal ontologies** can be used to standardise the specification of clinical goals. For example, in the Goal-based Comorbidity decision-support method [29], goals specification follows the goal ontology developed by Fox et al. [30] that includes a verb and a noun phrase (eg, manage hypertension, prevent cardiovascular disease, treat fatigue), the HL7 FHIR [14] Goal resource, and relationships from the National Drug File - Reference Terminology (NDF-RT) ontology [31], such as may-treat, may-prevent, and has_physiological_effect [increase/decrease State] e.g., Increase_Physical_Activity (NDF-RT Physiological Effect Goal). Alternatively, in the Asbru [27] clinical guideline formalism, process goals (eg, monitor blood pressure) or state goals (eg, normal blood pressure) can be specified as temporal patterns that are meant to be maintained, avoided or achieved (e.g., achieve systolic blood pressure <140 within 1 month of starting antihypertensive medication).

**Fig. 7:**
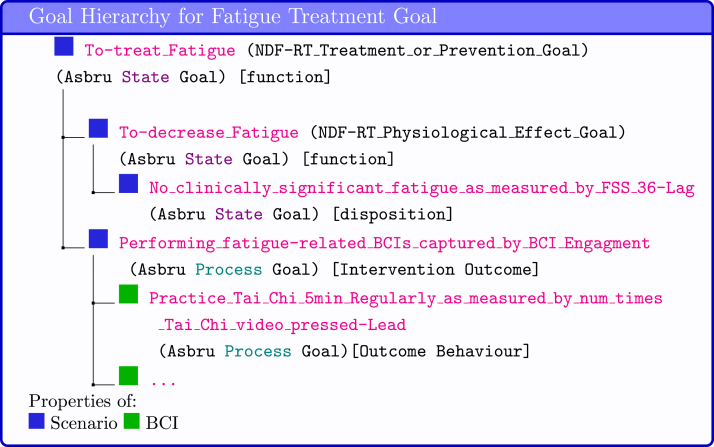
Case Study: Goal Hierarchy. The names of ontologies relating to clinical goals are shown in parentheses. BCIO concepts are shown in square brackets.

As BCIO follows the Basic Formal Ontology (BFO) [19], may-treat and may-prevent goals are represented as functions, and state goals (representing combination of lag measures and restrictions on them) are represented as dispositions. Finally, an Intervention Evaluation Study that evaluates a BCI Scenario (e.g., Fatigue Reduction Scenario) has output of Evaluation Finding (e.g. fatigue as measured by FSS). Related to the BCI, a BCI Evaluation Study that evaluates a BCI (e.g., Tai Chi BCI) has output of Evaluation Finding (e.g. number of times the Tai Chi video was pressed)(see Fig. 7).

#### 3.1.3 [S1.3] Define Lag Measures

To evaluate intervention effectiveness in supporting patients with reaching their clinical goals, researchers utilise standard patient-reported outcome measures (PROMs) [32] at the commencement and termination of the intervention. Each PROM should be selected to assess the patients’ states in dimension relevant to their clinical goals. Changes in scores on PROMs do not occur rapidly and therefore they are called lag measures [33].

**Fig. 8:**
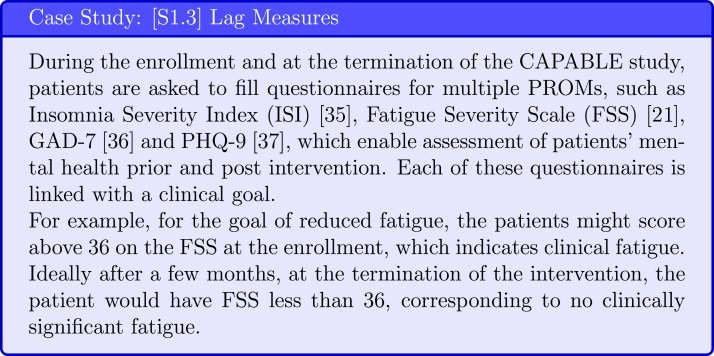
Case Study: Examples of lag measures [20], [34], [35], [36].

Note that for the BCI Scenario we measure the change in patients’ physiological/emotional state related to their clinical goal and the performance of the target behaviours is considered at the BCI level (see Table 1 and Fig. 8).

### 3.2 [S2] Identify BCIs for the clinical goals — What changes in behaviour will support resolution of the problem?

To refine the clinical goals and identify the interventions that can meet the goals, we can turn to clinical practice guidelines. Following the evidence-based medicine (EBM) movement, “clinical practice guidelines that we can trust” are defined as “statements that include recommendations intended to optimise patient care, that are informed by a systematic review of evidence and an assessment of the benefit and harms of alternative care options for a clinical condition” (clinical objective/goal) [37]. **Clinical practice guidelines** usually address a specific clinical condition. Unfortunately, most of the clinical guidelines refer to medication-based care options, and evidence for non-medication interventions is usually limited. However, non-pharmacological life-style, exercise, and psycho-behavioural interventions are a promising way to care for mental wellbeing, including for example, chronic pain [38] and fatigue [39], and include evidence grades [40] based on cohort studies, and in some cases on randomised controlled trials and meta-analyses, which provide a higher grade of evidence. Even though the non-pharmacological therapies are not widely spread there are some guidelines recommended by EBM such as ESMO fatigue guideline [39] and a back-pain guideline [38].

#### 3.2.1 [S2.1] Search for evidence-based intervention

**Fig. 9:**
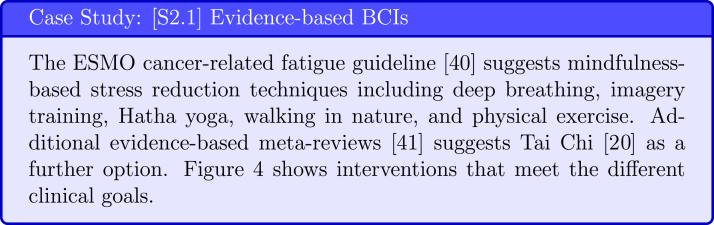
Case Study: Evidence-based BCIs [41].

Clinical goal(s) is an important extension of the BCIO and searching for evidence-based intervention options that meet it fits the “specify target behaviour” step of the Integrate phase of IDEAS. As mentioned above, clinical practice guidelines, and other clinical sources following the EBM pyramid, are the best resource to search for evidence-based interventions. We reused the BCIO’s BCI_Source property, which originally is a property of a BCT, and linked it to BCI to highlight that intervention choice should be supported by relevant evidence (See Table 1 and Fig. 9).

#### 3.2.2 [S2.2] Define Lead Measures

**Fig. 10:**
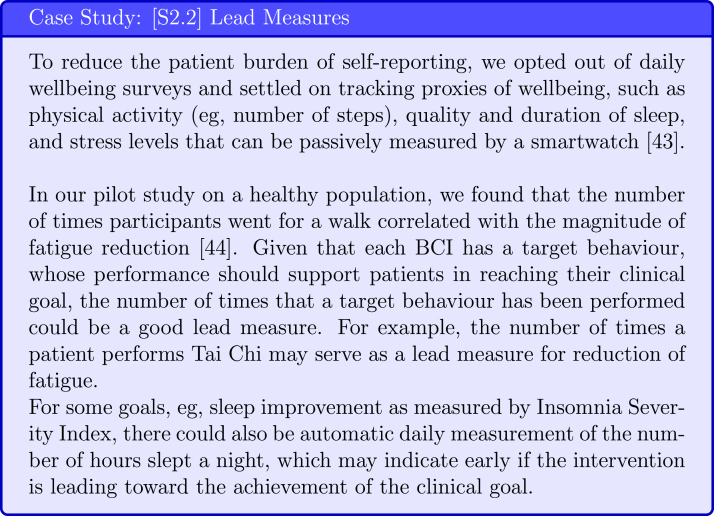
Case Study: Identifying and refining lead measures through pilot studies [43], [44].

To ensure that the BCI effectively supports patients in reaching the target clinical goal, ideally we would be able to check if a patient is on track of reaching their goal, and if not, modify the intervention. However, daily assessment through PROM questionnaires is not feasible long term, especially given that previous studies found that frequent surveys were not perceived favourable by the study participants [42] and could negatively impact engagement with the intervention. Therefore, it is important to identify measures that are related to the outcome but can also be captured frequently and automatically. The examples are provided in Fig. 10.

#### 3.2.3 [S2.3] Select BCI supporting technologies

**Fig. 11:**
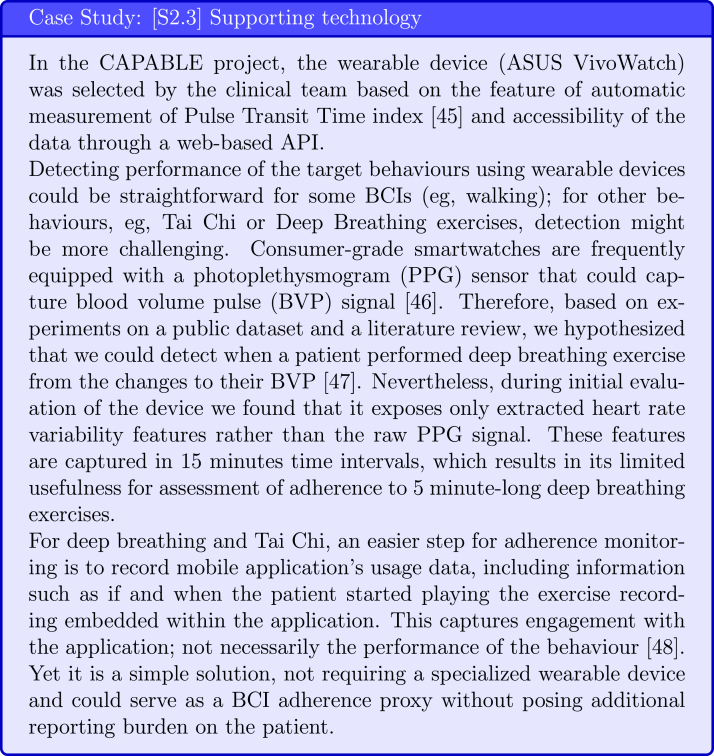
Case Study: Supporting technology selection and evaluation [45], [46], [47], [48].

To monitor intervention adherence, it might be helpful to pair the DBCI app with a wearable device. The choice of the device will depend on the population, their clinical goals, the selected BCIs and the lead measures. When selecting a wearable device we suggest to consider not only **type of captured data** but also the **frequency** and to test the candidate devices early in the application development cycle (see Fig. 11).

### 3.3 [S3] Create BCI Content — How can we facilitate patients with behaviour change?

Once the patient population, their context, clinical goals and evidence-based BCIs meeting the goals are identified from evidence-based sources, the development team use agile software development methods [21], including refinement of stereotypical persona and development of user stories, to create a shared understanding of the anticipated user experience with the DBCI. The goal of this step is to diagnose potential barriers to engagement with the intervention and determine which techniques could be used to overcome them.

#### 3.3.1 [S3.1] Create user stories

**Fig. 12:**
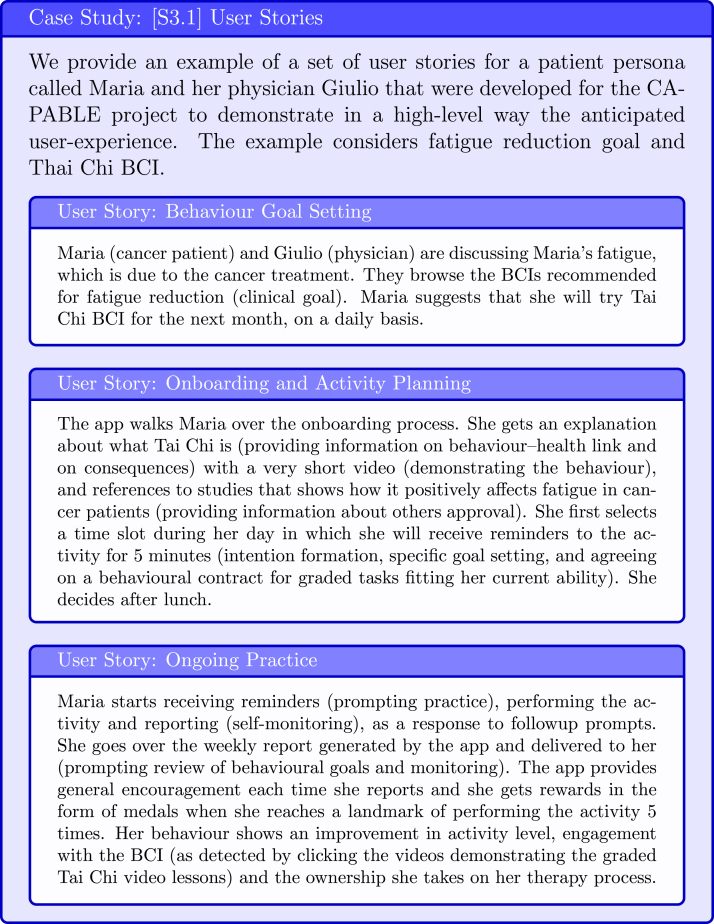
Case Study: User Stories.

The creation of user stories facilitate designers with identifying obstacles that patients may face with performing the target behaviour (see examples in Fig. 12). The user stories also form the starting point for app screen mockups, which are later further refined with users’ feedback.

#### 3.3.2 [S3.2] Find intervention protocols and source

**Fig. 13:**
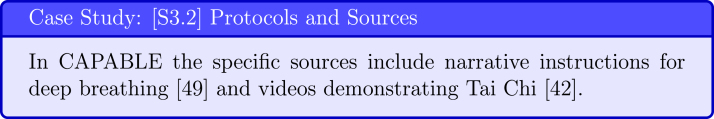
Case Study CAPABLE Project: Protocols and Sources [42], [49].

In further iterations of the Ideate step, the knowledge engineers in the team search for specific existing sources, i.e., implementations for the different interventions, in the form of narrative instructions or videos demonstrating behaviour (see examples referenced in Fig. 13). These should be accepted by the clinicians and patients of the multidisciplinary app-design team.

**Fig. 14:**
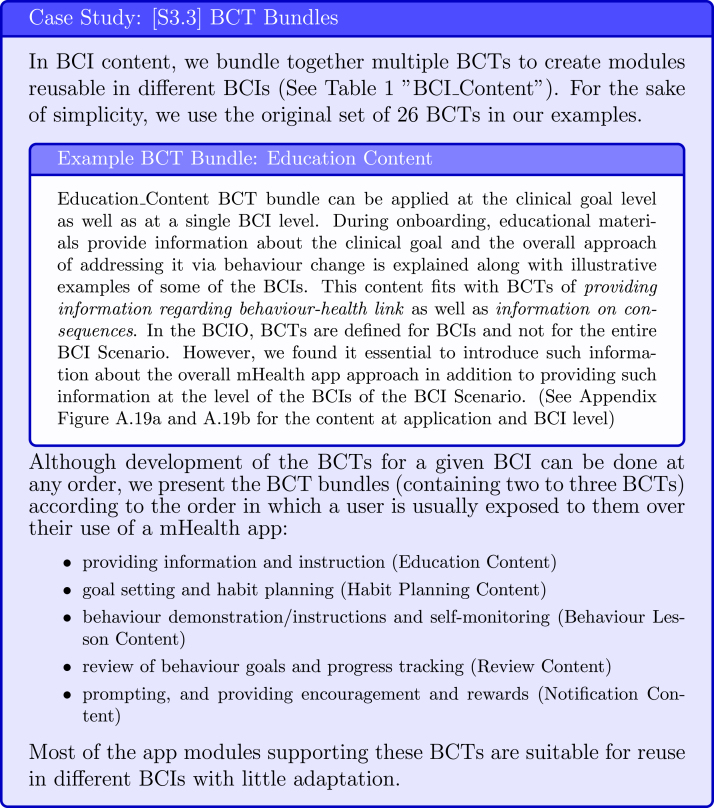
Case Stud: BCT Bundle templates.

#### 3.3.3 [S3.3] Select BCTs

Abraham and Michie [6] created a taxonomy of BCTs, which initially included 26 distinct BCTs and later was extended to 93 BCTs [50]. The most recent list includes 74 BCTs and is accompanied by a tool which helps to explore the links between the BCTs and mechanism of action https://theoryandtechniquetool.humanbehaviourchange.org/tool [51].

We suggest that BCTs could be bundled together to create BCI content and applied both on clinical goal and at single BCI levels (see Fig. 14). The BCT bundles organised into GUI designs fit with Ideate and Prototype steps from Design stage of IDEAS framework respectively.

### 3.4 [S4] Customise and Personalise — How can we adjust the support to meet the needs of each individual?

Customisation (or customisability) refers to a creation of a predefined set of options during the design step. The options, for example may include multiple BCIs contributing to the same goal, different levels of activity difficulty for varying levels of patient skill or sets of motivational messages addressing varying patients beliefs and needs.

Personalisation on the other hand, is a process of matching the best options to a given patient at run time. This can either be performed by the user or by an algorithms. Personalisation is one of the most commonly used techniques in mobile health interventions [52] and it plays important role in influencing their effectiveness [53].

Tong et al. [54] conducted a systematic review of personalised mobile BCIs and highlighted that personalisation might be applied to: BCI_Content (e.g., demonstration video), BCI_Mode_of_delivery (e.g., voice message, game, wearable), BCI_Dose (e.g., number of daily notifications), BCI_Schedule_of_delivery. This means that personalisation considers what information is presented, how, when, and how often. Automatic, data-driven personalisation depends also on the type and source of the collected data, the frequency of data collection, and the personalisation algorithm. These should be also already considered when defining a set of customisation options.

**Fig. 15:**
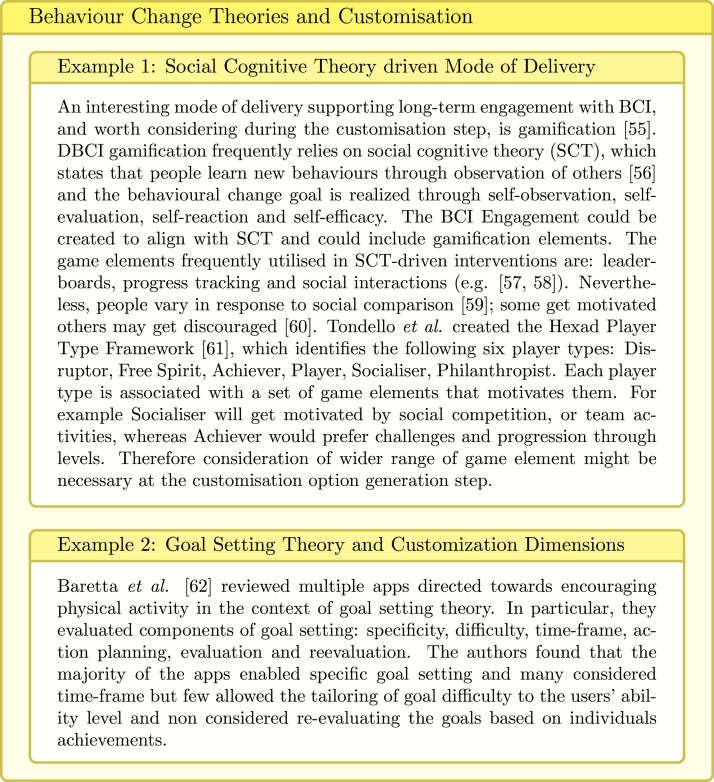
Behaviour Change Theories and BCI Tailoring [55], [56], [57], [58], [59], [60], [61], [62].

#### 3.4.1 [S4.1] Define a set of customisation options

**Fig. 16:**
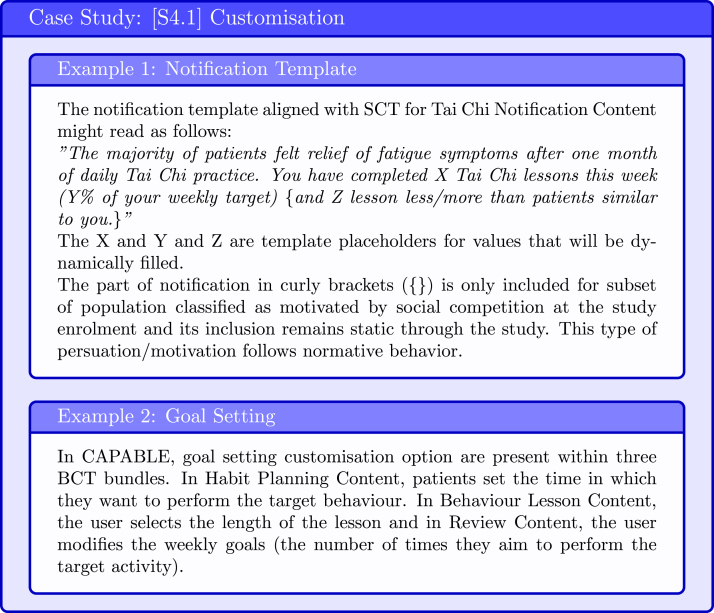
Case Study: Customisation.

To maximise the impact of education information on patients’ outcome behaviour, it is important that patients perceive the information to be personally relevant. Ghalibaf et al. conducted a systematic review of computer-based health information tailoring and identified six dimensions according to which patients could be characterised, these are: (1) socio-demographic (e.g., age, level of education), (2) medical history (eg, comorbidities), (3) health state (e.g., disease severity), (4) psycho-behavioural determinants (e.g., attitude, self-efficacy) (5) knowledge level, and (6) history of interactions (e.g., visited pages) [63]. In practice, the majority of studies used three or fewer dimensions for user profiling with socio-demographic and psycho-behavioural features being the most popular. The choice of the user categorisation dimensions depends on the BCI Content. Some parts are *static* (i.e., selected once prior to interventions commencement) for example patient name that is used in reminders; other are *dynamic* (i.e., change depending on the user interaction with the application) for example the content and phrasing of notification (See Fig. 16).

#### 3.4.2 [S4.2] develop personalisation methods

The goal of personalisation is to match the best available customisation option to the patient in order to maximise the probability that they perform the target behaviour. According to Fogg’s Behaviour Model (FBM) [64], three factors impact behaviour completion: motivation, ability and trigger. Tailoring of Notification Content and Education Content may increase patients’ motivation, Behaviour Lesson Dose (e.g. length of the thai chi lesson), the user’s ability to perform target behaviour, and schedule of prompt delivery the user’s responsiveness to the notification.

**Fig. 17:**
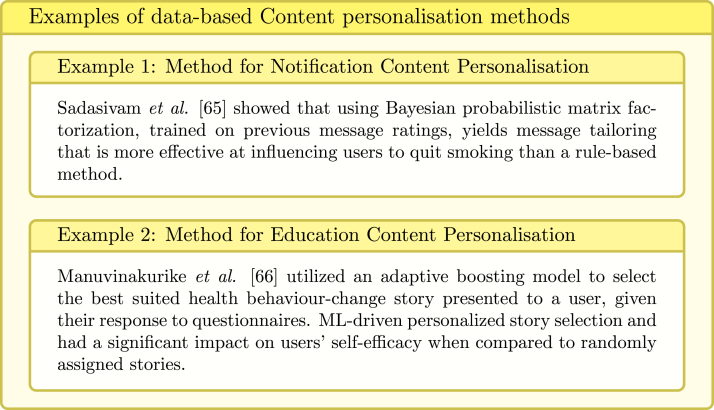
Examples of data-based Content personalisation [65], [66].

**Fig. 18:**
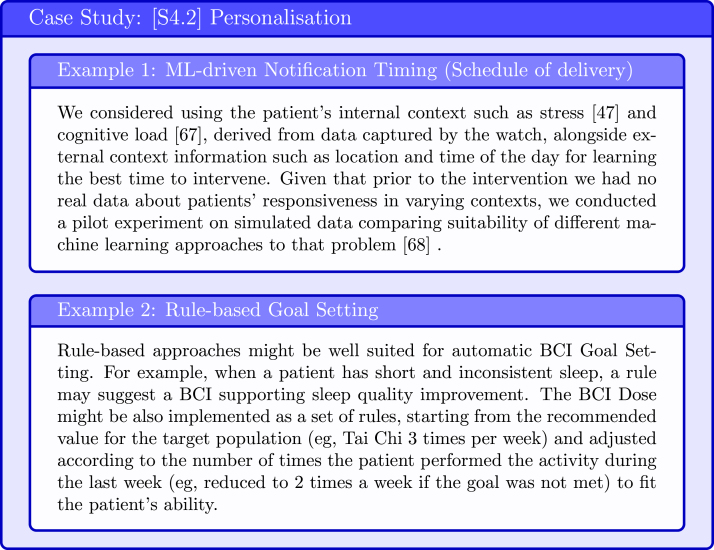
Case Study: Automatic Personalisation Methods [47], [67], [68].

The personalisation can be performed manually by the user or automatically by the system. The automatic personalisation algorithms are either knowledge-based or data-based. The former rely on human expertise and most commonly incorporate a set of rules that determine how the system should behave at various conditions. The later rely on data and might utilise machine learning (ML) models (see examples in Fig. 17, Fig. 18).

## 4 Evaluation

In the evaluation of the SATO workflow, we focused on: (1) demonstration of the utilisation of the workflow in the complex multi-BCIs mHealth app design, and on (2) assessment of the workflow’s clarity and its usefulness to the application design process. The former is achieved through CAPABLE app design (Section 4.1) and the latter is achieved through preliminary study with two knowledge engineers who were asked to design an application using the SATO workflow for a novel scenario (Section 4.2).

### 4.1 SATO validation for multiple BCIs

We evaluated the applicability of our workflow and checklist by considering multiple clinical goals/BCI Scenarios, multiple BCIs (Capsules), and BCT Bundles (see Fig. 4). We found that our methods supported design of application for all of the considered scenarios, BCIs and BCT bundles. In Table 2 we summarise goals and interventions for which we defined content as part of the CAPABLE app. In Section 3 and Table 1 we demonstrate examples from the fatigue reduction scenario; however the CAPABLE app targets a wider range of goals with seven different BCIs for which content was developed following the SATO workflow (See A Fig. A.20(b)).

**Table 2:** Goals and content defined in CAPABLE app.

	Names	Total number
Goals BCI Scenarios	Fatigue Reduction Sleep Improvement Stress/Anxiety Reduction Mood Improvement Physical Activity Increase	5

BCIs (Capsules)	Deep Breathing, Imagery Training, Tai Chi, Yoga, Garden Bowl, Gratitude Journal, Photo Collage	7

BCT Bundles	Education_Content Habit_Planning_Content Behaviour_Lesson_Content Review_Content Notification_Content	5

**Table 3:**
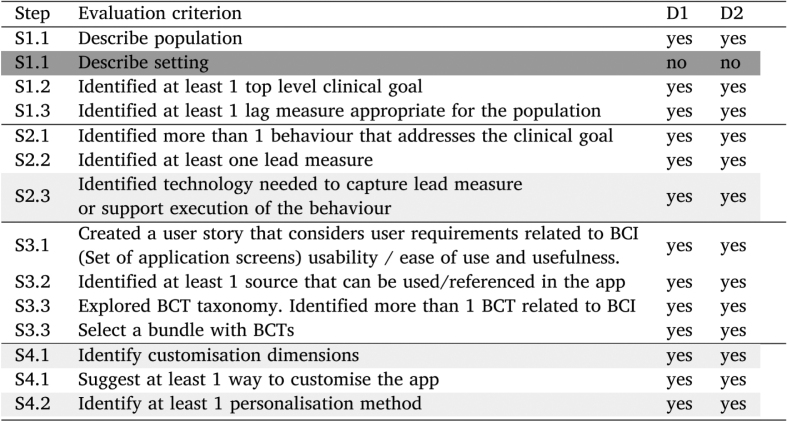
Evaluation results.

### 4.2 SATO clarity evaluation

To assess the clarity of the SATO workflow, we asked two knowledge engineers to use it to design a mobile behaviour change application. The engineers first participated in a short tutorial which walked them through the SATO workflow steps and provided examples of the step execution (the same as those presented in blue case study tables). Then they were given two papers [69], [70] serving as EBM sources and a very brief problem statement on the basis of which they were asked to complete each step in the checklist of the SATO workflow. The task was to design a mobile DBCI app for helping educators to prevent burnout (see B). The assessment criteria of the design were created prior to performing the task by the knowledge engineers. The evaluation criteria and results are presented in Table 3. The majority of the steps were performed very well; the steps which proved to be challenging are highlighted in grey and briefly discussed below.

Participants struggled the most with understanding of the BCI scenario context. Both participants correctly identified the population, but one participant skipped the description of the setting and the other redefined the population in the description of setting. To clarify this step we added in Section 3.1.1 a suggestion to create user personas to guide definition of both population and their setting.

**Fig. A.19:**
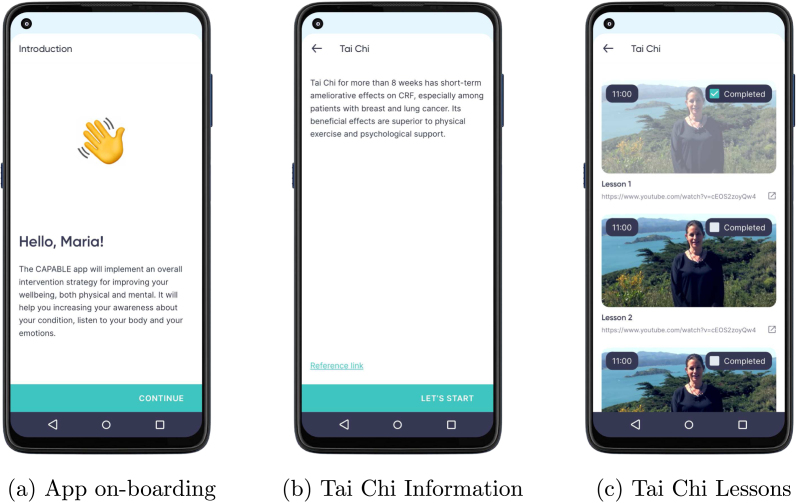
Mockup screens for education content at the application and individual BCI level. Mock-ups were created by Bitsens UAB, a partner of the CAPABLE Consortium.

**Fig. A.20:**
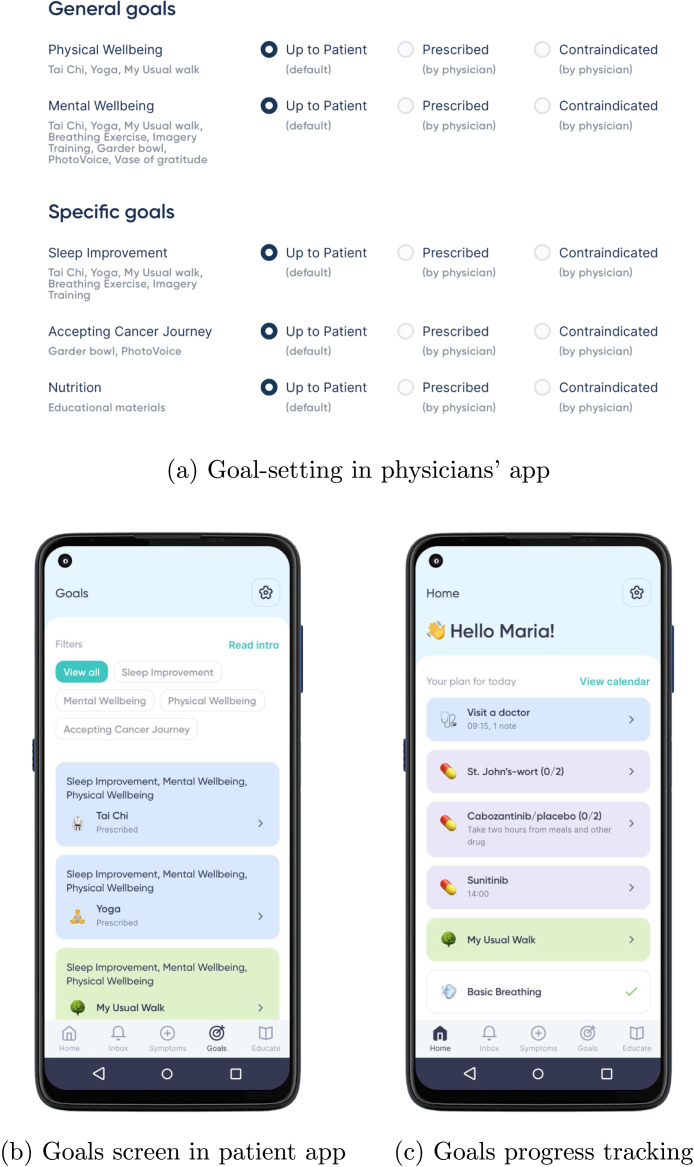
(a) Mockup of a goal-setting screen in the physicians’ app that is used during shared decision-making to set up goals for the patient (agree on a behavioural contract and review behavioural goals); (b) Mockup of the patient app showing BCIs. (c) Mockup of the goal-review screen with feedback on how many of the behaviour goals were achieved compared to the set target. Mock-ups were created by Bitsens UAB, a partner of the CAPABLE Consortium.

The other steps which might have been challenging for the participants was selection of supporting technology. Although both participants identified required technology the justification they provided for their selection was brief and did not consider reducing burden of self reporting through automation. At this step it might be already helpful to consider engagement data and the AMUsED framework [12].

The BCI tailoring step might also have been not perfectly clear. Both participants identified customisation options related to the schedule of delivery and content, but only one participant included in her design a customisation option for dose and mode of delivery. The two participants also used different approaches when selecting a personalisation method; one participant selected machine learning for content recommendation and described the data required for training of the model, wheres the other participant described manual personalisation of the schedule of delivery by the user. To improve the understanding of tailoring in Section 3.4 we highlighted in yellow boxes examples from the literature of utilising behaviour change theories in customisation (Fig. 15) and examples of developed personalisation algorithms (Fig. 17). These examples become part of the SATO tutorial (the workflow itself has not been modified).

## 5 Discussion

We proposed SATO, a DBCI design workflow aligned with the BCIO and the IDEAS framework, which we evaluated as being comprehensive for designing several BCI Scenarios for an mHealth app for cancer patients, as part of the CAPABLE project. To our knowledge, this is a first DBCI workflow which considers multiple BCIs and uses goal hierarchies with defined evaluation metrics at each level. We also provided examples on utilising behaviour change theories, such as SCT and FBM, when considering customisation and personalisation of the BCI for maximum engagement and adherence. The workflow and accompanying checklist were shown to be easy to follow when evaluated with two knowledge engineers in a novel application design scenario.

Although we presented the design steps consecutively, in practice they are iterative (see Fig. 3). For example, at the point of defining the target population and setting, we started creating the user stories for the entire app and later refined them for each BCI Scenario and BCI. Similarly, the lead measures were changed after testing the actual capabilities of the selected smartwatches. Moreover, the knowledge engineers on our team suggested to incorporate a wide range of BCTs; nevertheless some content elements (eg, providing feedback through progress visualisation) were not included in the final app. Specifically, the psychologists who were part of the multi-stakeholder development team raised concern that the ability of cancer patients to perform target behaviour might actually deteriorate with time due to the toxic effects of the cancer therapy and the course of illness; in such cases, visualising progress data might negatively impact their emotional well-being. This example highlights the need for iterative redesign with the end users being continuously kept in mind. In this context SATO aligns very closely with the Gather step of DEsign stage of IDEAS framework.

The proposed SATO workflow focuses strongly on the early phase of DBCI development, therefore the Share step from IDEAS framework is not comprehensively addressed. We have however utilised BCIO and extended it to facilitate knowledge sharing. The clinical evaluation study of the CAPABLE app, developed following SATO, with the cancer patients has not yet commenced, hence we could not provide concrete examples of Evaluation Findings. We will address this limitation and also evaluate the suitability of our chosen lead measures in future work, when conducting the clinical study of the usage of the CAPABLE system by cancer patients.

The IDEAS framework guides multi-disciplinary teams through mHealth apps development process. BCIO on the other hand captures knowledge in the behaviour change domain and introduces common language for the behaviour change theories aiming to support linking BCIO entities with evidence from behavioural studies. SATO builds on top of both and aims to translate knowledge represented within BCIO into the Integrate and DEsign stage of IDEAS. BCIO entities are directly coupled with the SATO workflow steps to facilitate mHealth app developers with utilising BCIO concepts. The SATO does not aim to replace IDEAS but rather complement its initial stages to ease future knowledge sharing and mHealth app evaluation in context of BCIs effectiveness.

## 6 Conclusions

We described a process of designing digital behaviour change intervention which incorporates a range of behaviour change techniques and addresses multiple clinical goals. The step-by-step SATO workflow that we created extends BCIO to support the scenarios with multiple intertwined and hierarchical goals and therefore could be used for design of any non-pharmacological digital behaviour change intervention. We aimed to keep the process simple and provide concrete examples of: technology-independent system captured lead measures, application modules bundling several BCTs, and customisation templates based on behaviour change theories which could be readily reused in other DBCIs.

## CRediT authorship contribution statement

**Aneta Lisowska:** Conceptualization, Methodology, Formal analysis, Investigation, Writing – original draft. **Szymon Wilk:** Conceptualization, Methodology, Formal analysis, Investigation, Writing – review & editing. **Mor Peleg:** Conceptualization, Validation, Investigation, Writing – review & editing, Project administration.

## Declaration of Competing Interest

The authors declare the following financial interests/personal relationships which may be considered as potential competing interests: Co-author Prof. Mor Peleg is Editor-in-Chief of Journal of Biomedical Informatics.

## Appendix A Mockups

See Fig. A.19.

## Appendix B Evaluation task

Follow SATO workflow to design mobile behaviour change intervention application for burnout educators.

Please use only the SATO workflow, provided burnout papers [69], [70] and https://theoryandtechniquetool.humanbehaviourchange.org/tool

Provide a short answer for each check mark in the SATO workflow. Ideally build a similar graph to one shown in the tutorial (see slide 35). Create BCI content for only one of identified BCIs. We are interested to evaluate if the workflow steps are clear and easy to follow not in the full fetched application design. Write down all applications aspects that can be customised and personalised and provide concrete examples how.

## Footnotes

1 extensions are marked with * in Table 1. We added goals and reused existing properties at different levels of the ontology to match the multi BCIs mHealth app requirements.
